# The association of *in-utero* exposure to air pollution and atherogenic index of plasma in newborns

**DOI:** 10.1186/s12940-024-01059-1

**Published:** 2024-02-19

**Authors:** Ali Seidkhani-Nahal, Hafez Heydari, Ayoub Tavakolian, Moslem Lari Najafi, Mohammad Miri

**Affiliations:** 1 Department of Clinical Biochemistry, Faculty of Medical Sciences, Ilam University of Medical Sciences, Ilam, Iran; 2 Department of Biochemistry, School of Medicine, Iran University of Medical Sciences, Tehran, Iran; 3 Emergency Department, Faculty of Medicine, Mashhad University of Medical Sciences, Mashhad, Iran; 4 Pharmaceutical Sciences and Cosmetic Products Research Center, Kerman University of Medical Sciences, Kerman, Iran; 5 Leishmaniasis Research Center, Department of Environmental Health, Sabzevar University of Medical Sciences, Sabzevar, Iran

**Keywords:** Air pollution, Atherogenic index, Cardiovascular diseases

## Abstract

**Background:**

Prenatal exposure to particulate matter (PM) and traffic was associated with the programming of cardiovascular diseases (CVDs) in early life. However, the exact underlying mechanisms are not fully understood. Therefore, we aimed to evaluate the association between *in-utero* exposure to PMs and traffic indicators with the atherogenic index of plasma (AIP) in newborns, which is a precise index reflecting an enhancement of lipid risk factors for CVDs.

**Methods:**

In this cross-sectional study, a total of 300 mother-newborn pairs were enrolled in Sabzevar, Iran. Spatiotemporal land-use regression models were used to estimate the level of PM_1_, PM_2.5_ and PM_10_ at the mother's residential address. The total length of streets in different buffers (100,300 and 500m) and proximity to major roads were calculated as indicators of traffic. The AIP of cord blood samples was calculated using an AIP calculator. Multiple linear regression models were used to examine the association of PM concentrations as well as traffic indicators with AIP controlled for relevant covariates.

**Results:**

PM_2.5_ exposure was significantly associated with higher levels of AIP in newborns. Each interquartile range (IQR) increment of PM_2.5_ concentration at the mothers' residential addresses was associated with a 5.3% (95% confidence interval (CI): 0.0, 10.6%, *P* = 0.04) increase in the AIP. Associations between PM_1_, PM_10_ and traffic indicators with cord blood level of AIP were positive but not statistically significant.

**Conclusion:**

Our findings showed that in utero exposure to PM_2.5_ may be associated with CVDs programming through the increase of atherogenic lipids.

**Supplementary Information:**

The online version contains supplementary material available at 10.1186/s12940-024-01059-1.

## Introduction

In recent decades, with the rapid growth of new-type urbanization, the risk of exposure to air pollution has increased [1–3]. Globally, the majority of urban residents (approximately 85%) are exposed to air pollution levels exceeding the safety guidelines established by the World Health Organization (WHO) [4, 5].

Prenatal exposure to particulate matter (PM) and traffic-related air pollution during pregnancy were associated with significant adverse changes in the programming of cardiovascular diseases (CVDs) [6–8]. The evidence reported that offspring born to mothers who have been exposed to high concentrations of PMs or traffic-related air pollution during pregnancy have a greater risk of developing CVDs in adulthood [6, 9]. Prospective studies confirm the association of prenatal exposure to PM or traffic-related air pollution with the risk of CVDs development [6, 10–12]. However, the molecular mechanisms that link maternal exposure to PMs and traffic-related air pollution to the programming of CVDs in offspring are not fully understood.

In an attempt to clarify the possible mechanisms, it is suggested that maternal exposure to PM or traffic leads to an increment of atherogenic lipids in the fetus [13, 14]. Elevated concentrations of atherogenic lipids such as cholesterol interfere with cardiovascular system development, leading to higher susceptibility to developing CVDs in future life [15]. Additionally, elevated atherogenic lipids induce stable epigenetic changes that increase the risk of CVDs development [15, 16].

However, there is limited evidence about the relationship between maternal exposure to PMs or traffic with lipid metabolism in the fetus. Previous studies have investigated the association of maternal exposure to PMs with triglyceride (TG), total cholesterol (TC), low-density lipoprotein cholesterol (LDL-C) and high-density lipoprotein cholesterol (HDL-C) concentrations in serum obtained from umbilical cord blood samples, which are influenced by maternal age at pregnancy, stress and diet [1, 14]. Such effects could sometimes lead to the misinterpretation of laboratory findings in pregnant women.

Atherogenic index of plasma (AIP), is a novel, simple, cost-effective, and high-sensitivity lipid index that represents the balance between atherogenic and anti-atherogenic lipids in plasma [17]. In this study, we employ the AIP as a robust metric for assessing lipid-related cardiovascular risk factors. While its evaluation in umbilical cord blood is relatively uncharted territory, our choice is underpinned by the AIP's well-established merits. Notably, AIP exhibits sensitivity and specificity in both pediatric and adult populations, surpassing traditional lipid indices and individual lipid parameters [18, 19]. What sets AIP apart are its inherent advantages, such as insensitivity to dietary influences and its proportional correlation with LDL particle size. Its role as a surrogate marker for small dense LDL (sdLDL) particles, which are notorious for their susceptibility to oxidation and atherosclerotic initiation [20, 21], underscores its value as a cardiovascular risk indicator. Moreover, the automatic logarithmic transformation feature inherent to AIP sets it apart, facilitating consistent log-transformed results an attribute not shared by conventional lipid profiles [20]. Thus, AIP emerges as a promising tool to unravel lipid-associated risk factors in our umbilical cord blood analysis [17]. Additionally, several comparative studies have reported the superiority of AIP over the measurement of lipid concentrations or conventional lipid ratios for the prediction of CVDs risk [22, 23]. Therefore, our aim in this study was to examine the association of *in-utero* exposure to air pollution (PM and traffic indicators) with the AIP in cord blood samples in Sabzevar, Iran.

## Materials and methods

### Study location

**Fig. 1:**
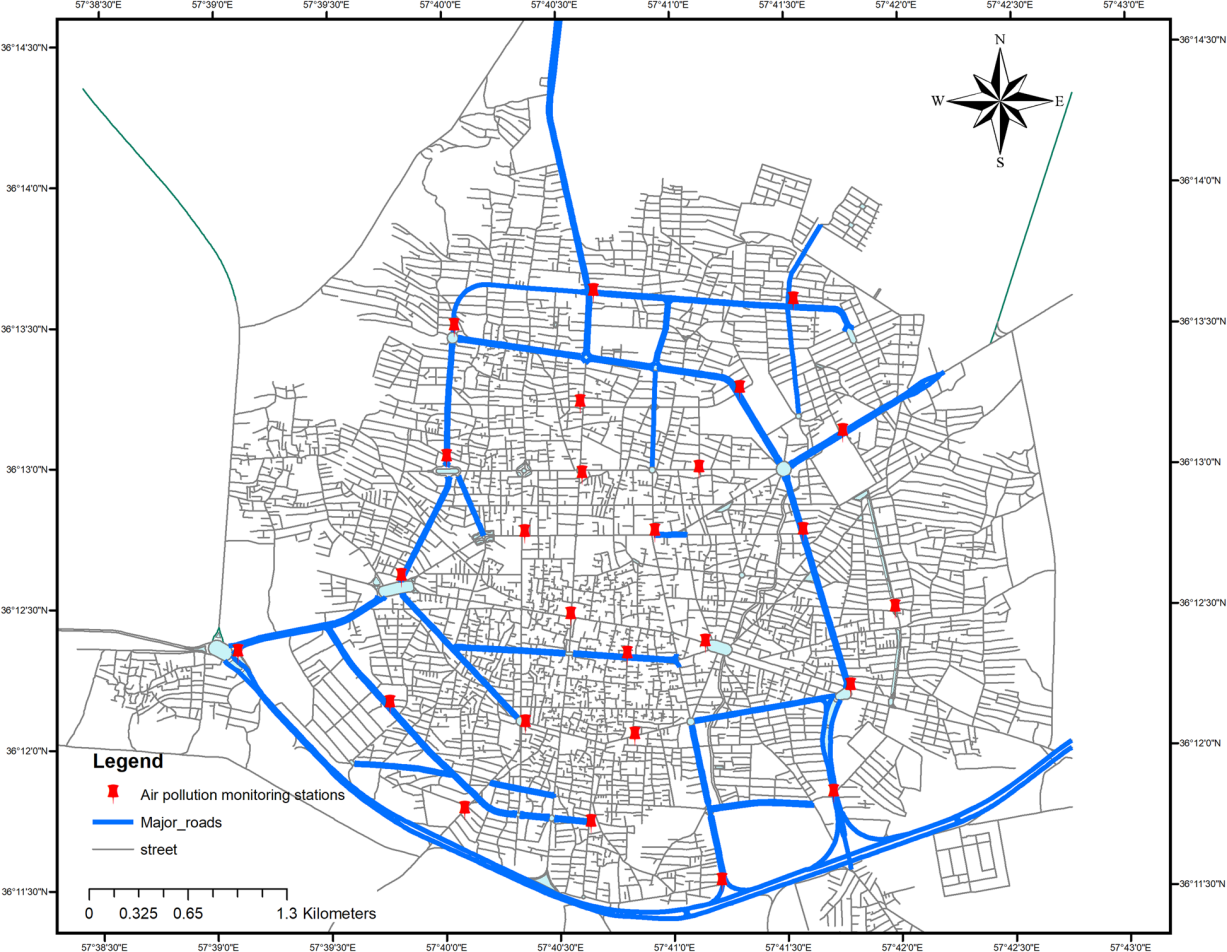
Study area including air pollution monitoring stations and roads

The current research was conducted in Sabzevar, a city located in the northeastern part of Iran (latitude: 36°12′54.66"N, longitude: 57°40′4, elevation: 977.6 m). According to the 2016 census, the city's population is estimated to be 240,000 people. Sabzevar has a dry climate with a relative humidity of 43%, annual mean precipitation of 176.8 mm and an annual average temperature of 16 °C. Figure 1 illustrates the research area, the location of air pollution monitoring stations, and the main roadways.

### Study population

This cross-sectional study was conducted on 300 pregnant women who were referred to Mobini Hospital (Sabzevar, Iran) for delivery between 2022 and 2023. Initially, 850 pregnant women were enrolled in the study, but only 300 met the inclusion criteria. The inclusion criteria were having a normal term pregnancy (37–42 weeks) and spontaneous vaginal delivery and did not work outside the home (i.e., they were housewives) while the exclusion criteria were diabetes, hypertension and pregnancy-induced disorders such as gestational diabetes and preeclampsia. Moreover, the women who changed their residence during pregnancy were excluded to maximise exposure estimation accuracy.

The lifestyle factors and socioeconomic status of pregnant women were collected through face-to-face interviews using a questionnaire. Before enrollment, the study objectives and steps were explained to participants who met the inclusion criteria, and informed consent was obtained from each participant. This study was approved by the Scientific & Ethics Committees of Ilam University of Medical Sciences (ethics code of IR.MEDILAM.REC.1401.203).

### Particulate matter (PM) exposures

Maternal exposure to PM_1_, PM_2.5_, and PM_10_ at residential addresses during the entire pregnancy was estimated using land-use regression (LUR) models developed for Sabzevar. The annual average was used to estimate exposure to PMs during pregnancy at the residential address. To develop the LUR models, PM concentrations were measured by a real-time monitoring device (HAZ-DUST EPAM 5000, USA) based on the United States Environmental Protection Agency (US EPA) method. The LUR model was established by analyzing 104 potential predictor variables characterized based on the geographic location of the monitoring station, population size, different land use, urban morphology, and traffic situation. The adjusted R^2^ of LUR models were 68%, 71%, and 75% for PM_1_, PM_2.5_, and PM_10_, respectively (Table S1 of Sublemental Materials). Further details of developed LUR models and PM measurements have been distributed elsewhere [24, 25].

### Traffic indicators

The information regarding the total length of streets within 100, 300, and 500-m zones around participants' residential locations, along with the proximity of residences to the nearest major roads, was derived from Sabzevar's street map, which was made available by the Sabzevar Municipality in 2019 and using ArcGIS software version 10.5. It should be noted that the major roads were classified in the street map of Sabzevar according to their functions and capacities (i.e. traffic volume).

### Serological measurements

Immediately after delivery, 4 ml of cord blood was obtained from all women and poured into tubes containing ethylenediamine tetraacetic acid (EDTA) to isolate plasma samples. To achieve this, the specimens were centrifuged at 3000 rpm at 4°C for 15 min. After separating the plasma samples, the concentrations of TAG and HDL-C were determined using available commercial kits with an auto-analyzer device. The atherogenic index was calculated using an online tool available at (http://www.biomed.cas.cz/fgu/aip/calculator.php). This online calculator estimates AIP using the following formula:

1 $${\text{AIP}}={\text{log}}\left({\text{TAG}}/{\text{HDL}}-{\text{C}}\right)$$

### Statistical analysis

#### Main analysis

In this study, multiple linear regression models were employed to evaluate the association between *in-utero* exposure to traffic indicators well as PM_1_, PM_2.5_, and PM_10_ as main independent variables (one at a time) and AIP (logarithmically transformed ratio) as a dependent variable (one at a time). Based on previous literature, we adjusted our model for relevant variables involved in our hypothesis including age (years, continuous) and the mother's body mass index (BMI) (kg/m^2^, continuous), exposure to second-hand smoke during pregnancy (Yes, No), household socioeconomic status (SES), including monthly income (Low, Middle, High), parental education (Elementary/illiterate, High-school, University) and neighbourhood SES, including the percentages of unemployment and illiterate adults per census tract. Regression coefficients were reported based on an interquartile range (IQR) increase in traffic indicators and concentrations of PM. The percentage change in AIP was calculated as (e^(β)^-1) × 100%, and 95% CI were calculated as (e^[(β±1.96_SE)]^-1) × 100%, in which SE is the estimated standard error and β is the estimated regression coefficient. The statistical analysis of the obtained data was carried out by STATA v.15 (Stata Corp LP, US) software, and the level of the statistical significance was set to *P* < 0.05.

#### Check other potential covariates

In order to examine whether the selected covariates have sufficient robustness, the model was further adjusted for the BMI values of infants, gender of infants (male/female), duration of exposure to smoke at home during pregnancy (minutes per day), exposure to tobacco smoke in public places other than home such as coffee shops and bus stations (yes/no), vehicle ownership (yes/no), homeownership (yes/no), use of a kitchen hood during cooking (yes/no) and the mean time spent cooking during pregnancy (minutes per day).

## Results

### Participant discription

Table 1 presents an overview of the descriptive statistics for the study participants. The median (IQR) age and pre-pregnancy BMI of mothers were 26 [7] years and 22.1 (6.3) kg/m^2^, respectively. Moreover, only 10.3% of women had a university education level. The median IQR of newborns' BMI was 12.1 (1.9) kg/m^2^and the median (IQR) of AIP in cord blood samples was -0.34 [26].

**Table 1:** Summary of participants' information, lipid profile as well as air pollution and traffic indicators data

Variables	Description
**Maternal characteristics**	
Age (year); median (first Q -third Q)	26 (23—30)
Pre-pregnancy BMI (kg/m2); median (first Q -third Q)	22.1 (20.2 – 26.5)
Education; N (%)	
Elementary/illiterate	87 (29.0%)
High-school	182 (60.6%)
University	31 (10.3%)
Self-reported tobacco exposure at home; N (%)	
Yes	56 (18.7%)
No	244 (81.3%)
Income; N (%)	
Low	169 (56.4%)
Middle	99 (33.0%)
High	32 (10.6%)
Census tract illiteracy; N (%)	27.5 (13.5–33.0)
Census tract unemployment; N (%)	7.1 (3.9–8.9)
**Newborn characteristics**; N (%)	
Gender	
Male	141 (47%)
Female	159 (53%)
BMI (kg/m2); median (first Q -third Q)	12.1 (11.5 – 13.4)
**Particulate matter (μg/m**^**3**^**)**; median (first Q -third Q)	
PM_1_	36.3 (29.5 – 65.3)
PM_2.5_	41.6 (35.1 – 74.1)
PM_10_	50.5 (40.3 – 84.5)
**Traffic indicators (m)**; median (first Q -third Q)	
Street length in a 100 m buffer	745(623 – 1231)
Street length in a 300 m buffer	8134 (5987 – 9271)
Street length in a 500 m buffer	21,067 (13,576 – 24,522)
Distance to major roads	196 (89 – 478)
**TAG (mg/dL)**; median (first Q -third Q)	31.3 (24.5 – 47.7)
**HDL-C (mg/dL)**; median (first Q -third Q)	28.9 (23.1 – 37.5)
**LDL-C (mg/dL)**; median (first Q -third Q)	23.7 (13.4 – 33.5)
**TC (mg/dL)**; median (first Q -third Q)	66.4 (49.5 – 74.4)
**AIP**; median (first Q -third Q)	-0.34 (-0.50 – -0.16)

*TAG* Triglyceride, *TC* Total cholesterol, *HDL-C* High-density lipoprotein cholesterol, *LDL-C* Low-density lipoprotein cholesterol, *AIP* Atherogenic index of plasma

Categorical variables are presented as count (%) and continuous variables as median (first quartile-third quartile)

The median (IQR) of estimated PM_1_, PM_2.5_, and PM_10_ concentrations at the maternal residential address were estimated to be 36.3 (35.8), 41.6 (39.0), and 50.5 (44.1) μg/m^3^, respectively. The median of total length of streets in 100, 300 and 500 m buffers was 745, 8134 and 21067 m, respectively.

According to the analysis of the Spearman Correlation Coefficient, a significant correlation was found between the concentrations of PM_1_ and PM_10_ (*r* = 0.79), PM_1_ and PM_2.5_ (*r* = 0.93), and PM_2.5_ and PM_10_ (*r* = 0.86). Moreover, there was a strong correlation between the total length of streets in the 100 m buffer with PM_1_, PM_2.5_ and PM_10_ (*r* = 0.69 – 0.86).

### Main analysis

Table 2 depicts the percentage change of AIP related maternal exposure to traffic indicators and PM concentrations. Based on the results obtained from the fully adjusted models, higher maternal exposure to PM_2.5_ during pregnancy was significantly associated with higher AIP in cord blood. Correspondingly, an increment of one IQR in the concentrations of PM_2.5_ was associated with 5.3% (95% confidence interval (CI): 0.00, 10.6%, *P* = 0.04) increase in AIP. In addition, higher concentrations of PM_1_ and PM_10_ were associated with higher values of AIP in the umbilical cord blood; however, these associations were not statistically significant. Moreover, higher levels of total length of streets in the 100 and 300-m buffers were positively associated with AIP, but these associations were not statistically significant. Furthermore, a higher distance from major roads was negatively but not significantly associated with AIP levels (Table 2).

**Table 2:** Association of exposure to PMs as well as traffic indicators and AIP

	**Crude**		**Adjusted**^a^	
**Exposure**	**Percentage chage (95% CI)**	***P*-value**	**Percentage chage (95% CI)**	***P*-value**
PM_1_	1.8 (-0.1, 3.7)	0.060	1.3 (-0.8, 3.4)	0.228
PM_2.5_	3.1 (-1.9, 8.1)	0.220	5.3 (0.00, 10.6)	0.049
PM_10_	3.5 (-1.3, 8.3)	0.155	4.6 (-0.5, 9.6)	0.075
Street length in a 100 m buffer	0.4 (-1.7, 2.5)	0.718	0.8 (-1.7, 3.3)	0.535
Street length in a 300 m buffer	1.5 (-3.5, 6.4)	0.559	0.4 (-5.0, 5.3)	0.882
Street length in a 500 m buffer	0.3 (-5.9, 6.4)	0.930	-1.2 (-7.8, 5.4)	0.724
Distance to major roads	-0.2 (-4.3, 3.9)	0.921	-1.7 (-6.0, 2.5)	0.421

*AIP* Atherogenic index of plasma, *PM* Particulate matter

Percentage changes were reported based on an interquartile range (IQR) increase in traffic indicators and concentrations of PM

^a^Models were adjusted for age and the mother's body mass index (BMI), exposure to second-hand smoke during pregnancy, income, parental education, percentages of unemployment and illiterate adults per census tract

### Other potential covariates

The results of regression models further adjusted for potential confounders, including BMI of infants, gender of infants, duration of exposure to smoke at home during pregnancy, exposure to tobacco smoke in public places other than home, vehicle ownership, homeownership, use of a kitchen hood during cooking, and the mean time spent cooking during pregnancy indicated no notable difference in term of significance and direction (Supplemental Materials Table S2-S9).

## Discussion

According to the relevant literature published so far, the current research is the first study to investigate the association of maternal exposure to PMs during pregnancy and traffic indicators with AIP in newborns. Furthermore, our study provides compelling evidence of the relationship between prenatal exposure to air pollution and lipid metabolism in newborns in low- and middle-income countries. We found a positive association between exposure to PM_2.5_ during pregnancy and higher levels of AIP in cord blood samples after adjusting for individual, demographic, lifestyle and socioeconomic factors. Although there was a positive association between exposure to PM_1_ and PM_10_ as well as street length in 100 and 300-m buffers, these associations were not statistically significant.

### Interpretation of the results

We did not find any studies that had investigated the association between maternal exposure to PMs during pregnancy and traffic indices with AIP in newborns. Therefore, it is not possible to compare our results with the previous evidence. However, our findings align with previous studies that have suggested the atherogenic effects of air pollution. For instance, Heydari et al*.* (2020) evaluated the association between maternal exposure to PMs and traffic-related air pollution and atherogenic lipids concentrations (i.e., TC and TAG, as well as the ratios of TG/HDL-C and TC/HDL-C) in cord blood samples [14]. They reported that maternal exposure to PM_2.5_ and PM_10_ during pregnancy was positively associated with TC, TAG, TC/HDL-C and TG/HDL-C. They also reported that the total length of streets in a buffer of 100m at the proximity of residential locations was positively associated with TC, LDL-C, HDL-C and TAG concentrations as well as the TC/HDL-C ratio [14].

Additionally, our results are in line with the findings of previous studies that investigated the association of long-term exposure to PMs and serum lipid concentrations in other population settings. For example, McGuinn et al., 2020 conducted a cohort study on 465 mother–child pairs and reported that PM_2.5_ exposure during the third trimester was associated with increases in childhood TC, LDL-C, and decreases in HDL-C [27]. Another study carried out by Yitshak et al*.* on 73 117 subjects, demonstrated that moderate-term exposure to PM_10_ was associated with decreased HDL-C in adults [28]. A similar study conducted on 2289 middle-aged women showed that long-term exposure to PM_2.5_ (annual average) was linked with increased concentrations of apolipoprotein A1, lipoprotein a, and HDL-C [29]. McGuinn et al*. *(2016) also conducted a study on 6587 patients with CVDs and found that long-term exposure to PM_2.5_ was markedly associated with increased concentrations of atherogenic lipids including TAG, TC, apolipoprotein B, and LDL-C [30]. Finally, Yang et al*.* (2018) conducted a population-based study on 15477 Chinese individuals with an age range of 18–74 years and showed that long-term exposure to PM_1_ and PM_2.5_ was significantly linked with an increase in LDL-C, TAG, and TC as well as reduced levels of HDL-C [31].

### Biological relevance

The precise mechanism underlying the changes in fetal lipid metabolism in response to maternal exposure to PMs is still unclear; however, a number of possible mechanisms have been suggested. First, maternal exposure to PMs increases proinflammatory cytokines production by immune cells [32]. Elevated concentrations of these cytokines decrease TAG clearance and increase VLDL synthesis [33]. Second, it has been shown that maternal exposure to high concentrations of PMs during pregnancy induces insulin resistance in the fetus [26]. Insulin resistance is associated with altered lipid metabolism (such as increased TAG and decreased HDL-C), which can lead to an increase in AIP [34]. Third, AIP changes may be due to epigenetic changes in genes affecting lipid metabolism in the fetus [15]. It has been shown that PMs can affect the expression of genes through epigenetic changes [15]. Several lines of evidence demonstrated the ability of PMs to cross the placenta and their influence on gene expression patterns via epigenetic modifications [15]. Fourthly, modification of lipid metabolism may be due to changes in hypothalamic–pituitary–adrenal (HPA) activity in the fetus. Sun and colleagues indicated the altered lipid metabolism in response to air pollution though affecting the hypothalamic–pituitary–adrenal (HPA) axis [35, 36]. Our results of associations of prenatal exposure to PMs and AIP in newborns could be explained by at least one of these mechanisms.

### Strength and limitations

The strength of our study include the use of highly sensitive and precise indices for evaluating of atherogenic lipids concentration (i.e. AIP) and adjusting for many potential covariates. However, our study had some limitations. We describe the association between AIP and maternal exposure to PMs; however, it is difficult to relate these findings to clinical consequences. Additionally, LUR models were used to analyze the association of maternal exposure to PMs with AIP levels, which may lead to potential exposure misclassification. Moreover, for our analyses, we assessed and applied participants’ exposure to outdoor air pollution at their residential address; however, pregnant women spend much of their time inside home where the air pollution levels could be different from those of the outdoor environment. Such an exposure assessment could have resulted in some degrees of exposure misclassification [37]. The indoor upon outdoor ratios of PM can be impacted by the air filtration systems [38], the number of occupants and their behaviours (like smoking), and building types [39, 40]. Hence, the measured outdoor PM levels may or may not be representative of what those women were exposed to during the pregnancy period. Furthermore, we did not have information about women’s preconception exposure to PMs, which could influence the outcome of our analysis. Also, physical activity during pregnancy may affect the concentrations of lipids in the umbilical cord, but we did not have information about the physical activity of mothers during pregnancy. One notable limitation of this study is the relatively small sample size, which may have contributed to the null findings observed in certain associations. The limited number of participants could have reduced the statistical power of the analysis, potentially hindering our ability to detect more subtle relationships between prenatal exposure to air pollutants and AIP. While we employed rigorous methodologies and adjusted for relevant covariates, the study's sample size remains a constraint in drawing definitive conclusions, and further research with larger cohorts could provide more robust insights into these associations.

## Conclusion

We observed that maternal exposure to PM_2.5_ was positively associated with the AIP in cord blood. Associations between PM_1_, PM_10_ and traffic indicators with cord blood level of AIP were positive but not statistically significant. If our findings are validated through future studies, they have the potential to provide valuable insights into the negative health implications of prenatal air pollution exposure, specifically, the risk of CVDs development in adulthood. Further longitudinal studies with larger population sizes are needed to clarify the exact biological mechanisms of AIP increase in response to PM_2.5_ exposure.

### Supplementary Information

**Additional file 1: Table S1.** The predictor variables and performance indicators of developed land use regression (LUR) models of annual mean PMs.** Table S2. **Regression coefficients of exposure to PMs as well as traffic indicators and AIP further adjusted for BMI values of infants.** Table S3. **Regression coefficients of exposure to PMs as well as traffic indicators and AIP further adjusted for gender of infants.** Table S4. **Regression coefficients of exposure to PMs as well as traffic indicators and AIP further adjusted for the duration of exposure to smoke at home during pregnancy.** Table S5. **Regression coefficients of exposure to PMs as well as traffic indicators and AIP further adjusted for exposure to tobacco smoke in public places other than home such as coffee shops and bus stations.** Table S6. **Regression coefficients of exposure to PMs as well as traffic indicators and AIP further adjusted for home ownership.** Table S7. **Regression coefficients of exposure to PMs as well as traffic indicators and AIP further adjusted for the use of a kitchen hood during cooking.** Table S8. **Regression coefficients of exposure to PMs as well as traffic indicators and AIP further adjusted for the mean time spent cooking during pregnancy.** Table S9. **Regression coefficients of exposure to PMs as well as traffic indicators and AIP further adjusted for car ownership.

## Data Availability

The data are available from the corresponding author upon reasonable request.
