# Supplementary material for: The association of in-utero exposure to air pollution and atherogenic index of plasma in newborns
Source: Environ Health. 2024 Feb 19;23:22. doi: 10.1186/s12940-024-01059-1 (PMC10875836; doi:10.1186/s12940-024-01059-1)
Supplement: Supplementary file 1 — Additional file 1: Table S1. The predictor variables and performance indicators of developed land use regression (LUR) models of annual mean PMs. Table S2. Regression coefficients of exposure to PMs as well as traffic indicators and AIP further adjusted for BMI values of infants. Table S3. Regression coefficients of exposure to PMs as well as traffic indicators and AIP further adjusted for gender of infants. Table S4. Regression coefficients of exposure to PMs as well as traffic indicators and AIP further adjusted for the duration of exposure to smoke at home during pregnancy. Table S5. Regression coefficients of exposure to PMs as well as traffic indicators and AIP further adjusted for exposure to tobacco smoke in public places other than home such as coffee shops and bus stations. Table S6. Regression coefficients of exposure to PMs as well as traffic indicators and AIP further adjusted for home ownership. Table S7. Regression coefficients of exposure to PMs as well as traffic indicators and AIP further adjusted for the use of a kitchen hood during cooking. Table S8. Regression coefficients of exposure to PMs as well as traffic indicators and AIP further adjusted for the mean time spent cooking during pregnancy. Table S9. Regression coefficients of exposure to PMs as well as traffic indicators and AIP further adjusted for car ownership. [file 12940_2024_1059_MOESM1_ESM.docx]

**Supplemental Materials**

The Association of *in-utero* Exposure to Air Pollution and Atherogenic Index of Plasma in Newborns

Ali Seidkhani-Nahal^1^, Hafez Heydari^2^*, [Ayoub Tavakolian](https://pubmed.ncbi.nlm.nih.gov/?term=Tavakolian+A&cauthor_id=36062028)^3^, Moslem Lari Najafi^4^ and Mohammad Miri*

**Table S1 ……………………………………………………………..page 2**

**Table S1:** The predictor variables and performance indicators of developed land use regression (LUR) models of annual mean PMs.

| **Pollutant** | **Equation** | **R^2^** | **R** | **RMSE** | **LOOCV**  **RMSE** | **Max VIF (variable)** |
| --- | --- | --- | --- | --- | --- | --- |
| PM_1_ | 33.52 + 2.12E-02* **DIST to RelCu** + 2.66E-03* **Ind_500** -1.29E-04* **Edu_500** | 0.68 | 0.82 | 5.21 | 6.18 | 6.40  (Edu_500) |
| PM_2.5_ | 41.70 + 3.28E-03* **Ind_500** + 2.30E-02* **DIST to RelCu** - 0.62***MH_200** | 0.71 | 0.95 | 3.33 | 4.7 | 3.56  (MH_200) |
| PM_10_ | 53.39 + 1.77E-03***Othr_100** + 4.05E-02* **DIST to RelCu** -4.14E-03* **DIST to BuT -** 9.52E-03* **DIST to UrFa** | 0.75 | 0.86 | 8.99 | 10.34 | 4.49  (Othr_100) |

**Note: DIST to BuT** = distance to bus terminal**; DIST to RelCu** = distance to religion/cultural land use**; DIST to UrFa =** distance to urban facility land use**; Edu** = education land use area; **Ind** = industrial land use; **MH =** maximum height of building**; Othr** = other land use area.

**Table S 2.** Regression coefficients of exposure to PMs as well as traffic indicators and AIP further adjusted for BMI values of infants

| Exposure | β-coefficient (95% CI) | P_value |
| --- | --- | --- |
| **PM pollutants** |  |  |
| PM_10_ | 0.014 (-0.009, 0.036) | 0.248 |
| PM_2.5_ | 0.063 (0.006, 0.126) | 0.040 |
| PM_1_ | 0.041 (-0.006, 0.090) | 0.083 |
| **Traffic indicators** |  |  |
| Street length in a 100 m buffer | 0.008 (-0.017, 0.033) | 0.549 |
| Street length in a 300 m buffer | 0.009 (-0.08, 0.067) | 0.910 |
| Street length in a 500 m buffer | -0.015 (-0.085, 0.069) | 0.831 |
| Distance to major roads | -0.021 (-0.08, 0.029) | 0.241 |

**Table S 3.** Regression coefficients of exposure to PMs as well as traffic indicators and AIP further adjusted for gender of infants

| Exposure | β-coefficient (95% CI) | P_value |
| --- | --- | --- |
| **PM pollutants** |  |  |
| PM_10_ | 0.011 (-0.007, 0.033) | 0.103 |
| PM_2.5_ | 0.051 (0.001, 0.101) | 0.048 |
| PM_1_ | 0.047 (-0.008, 0.099) | 0.065 |
| **Traffic indicators** |  |  |
| Street length in a 100 m buffer | 0.009 (-0.019, 0.035) | 0.549 |
| Street length in a 300 m buffer | 0.006 (-0.03, 0.055) | 0.182 |
| Street length in a 500 m buffer | -0.015 (-0.089, 0.034) | 0.224 |
| Distance to major roads | -0.018 (-0.08, 0.015) | 0.133 |

**Table S 4.** Regression coefficients of exposure to PMs as well as traffic indicators and AIP further adjusted for the duration of exposure to smoke at home during pregnancy

| Exposure | β-coefficient (95% CI) | P_value |
| --- | --- | --- |
| **PM pollutants** |  |  |
| PM_10_ | 0.012 (-0.009, 0.035) | 0.322 |
| PM_2.5_ | 0.065 (0.020, 0.124) | 0.038 |
| PM_1_ | 0.056 (-0.004, 0.117) | 0.060 |
| **Traffic indicators** |  |  |
| Street length in a 100 m buffer | 0.015 (-0.008, 0.043) | 0.175 |
| Street length in a 300 m buffer | 0.005 (-0.03, 0.049) | 0.782 |
| Street length in a 500 m buffer | -0.011 (-0.070, 0.053) | 0.432 |
| Distance to major roads | -0.019 (-0.09, 0.020) | 0.121 |

**Table S 5.** Regression coefficients of exposure to PMs as well as traffic indicators and AIP further adjusted for exposure to tobacco smoke in public places other than home such as coffee shops and bus stations

| Exposure | β-coefficient (95% CI) | P_value |
| --- | --- | --- |
| **PM pollutants** |  |  |
| PM_10_ | 0.014 (-0.009, 0.036) | 0.225 |
| PM_2.5_ | 0.054 (0.001, 0.107) | 0.048 |
| PM_1_ | 0.048 (-0.003, 0.099) | 0.070 |
| **Traffic indicators** |  |  |
| Street length in a 100 m buffer | 0.009 (-0.015, 0.035) | 0.530 |
| Street length in a 300 m buffer | 0.005 (-0.03, 0.055) | 0.875 |
| Street length in a 500 m buffer | -0.015 (-0.083, 0.059) | 0.729 |
| Distance to major roads | -0.019 (-0.08, 0.029) | 0.429 |

**Table S 6.** Regression coefficients of exposure to PMs as well as traffic indicators and AIP further adjusted for home ownership

| Exposure | β-coefficient (95% CI) | P_value |
| --- | --- | --- |
| **PM pollutants** |  |  |
| PM_10_ | 0.012 (-0.009, 0.035) | 0.231 |
| PM_2.5_ | 0.054 (0.00, 0.107) | 0.049 |
| PM_1_ | 0.049 (-0.008, 0.095) | 0.073 |
| **Traffic indicators** |  |  |
| Street length in a 100 m buffer | 0.009 (-0.018, 0.035) | 0.536 |
| Street length in a 300 m buffer | 0.005 (-0.06, 0.050) | 0.885 |
| Street length in a 500 m buffer | -0.015 (-0.079, 0.053) | 0.720 |
| Distance to major roads | -0.016 (-0.09, 0.023) | 0.415 |

**Table S 7.** Regression coefficients of exposure to PMs as well as traffic indicators and AIP further adjusted for the use of a kitchen hood during cooking

| Exposure | β-coefficient (95% CI) | P_value |
| --- | --- | --- |
| **PM pollutants** |  |  |
| PM_10_ | 0.014 (-0.005, 0.039) | 0.112 |
| PM_2.5_ | 0.059 (0.003, 0.117) | 0.034 |
| PM_1_ | 0.049 (-0.002, 0.099) | 0.055 |
| **Traffic indicators** |  |  |
| Street length in a 100 m buffer | 0.012 (-0.011, 0.039) | 0.115 |
| Street length in a 300 m buffer | 0.007 (-0.02, 0.071) | 0.182 |
| Street length in a 500 m buffer | -0.002 (-0.008, 0.079) | 0.233 |
| Distance to major roads | -0.019 (-0.07, 0.028) | 0.221 |

**Table S 8.** Regression coefficients of exposure to PMs as well as traffic indicators and AIP further adjusted for the mean time spent cooking during pregnancy

| Exposure | β-coefficient (95% CI) | P_value |
| --- | --- | --- |
| **PM pollutants** |  |  |
| PM_10_ | 0.011 (-0.006, 0.036) | 0.220 |
| PM_2.5_ | 0.051 (0.001, 0.109) | 0.040 |
| PM_1_ | 0.044 (-0.004, 0.099) | 0.070 |
| **Traffic indicators** |  |  |
| Street length in a 100 m buffer | 0.007 (-0.015, 0.039) | 0.633 |
| Street length in a 300 m buffer | 0.003 (-0.03, 0.058) | 0.880 |
| Street length in a 500 m buffer | -0.013 (-0.079, 0.059) | 0.720 |
| Distance to major roads | -0.018 (-0.08, 0.029) | 0.439 |

**Table S 9.** Regression coefficients of exposure to PMs as well as traffic indicators and AIP further adjusted for car ownership

| Exposure | β-coefficient (95% CI) | P_value |
| --- | --- | --- |
| **PM pollutants** |  |  |
| PM_10_ | 0.015 (-0.007, 0.036) | 0.221 |
| PM_2.5_ | 0.055 (0.001, 0.108) | 0.041 |
| PM_1_ | 0.047 (-0.002, 0.097) | 0.072 |
| **Traffic indicators** |  |  |
| Street length in a 100 m buffer | 0.005 (-0.018, 0.035) | 0.533 |
| Street length in a 300 m buffer | 0.003 (-0.03, 0.055) | 0.880 |
| Street length in a 500 m buffer | -0.013 (-0.070, 0.058) | 0.720 |
| Distance to major roads | -0.018 (-0.08, 0.029) | 0.415 |
